# Perturbed Biochemical Pathways and Associated Oxidative Stress Lead to Vascular Dysfunctions in Diabetic Retinopathy

**DOI:** 10.1155/2019/8458472

**Published:** 2019-03-06

**Authors:** Nidhi Mahajan, Palkin Arora, Rajat Sandhir

**Affiliations:** Panjab University, Department of Biochemistry, Basic Medical Science Block II, Chandigarh 160014, India

## Abstract

Diabetic retinopathy (DR) is a vascular insult that accompanies the hyperglycemic state. Retinal vasculature holds a pivotal role in maintaining the integrity of the retina, and any alteration to retinal vasculature affects retinal functions. The blood retinal barrier, a prerequisite to vision acuity, is most susceptible to damage during the progression of DR. This is a consequence of impaired biochemical pathways such as the polyol, advanced end glycation products (AGE), hexosamine, protein kinase C (PKC), and tissue renin-angiotensin system (RAS) pathways. Moreover, the role of histone modification and altered miRNA expression is also emerging as a major contributor. Epigenetic changes create a link between altered protein function and redox status of retinal cells, creating a state of metabolic memory. Although various biochemical pathways underlie the etiology of DR, the major insult to the retina is due to oxidative stress, a unifying factor of altered biochemical pathways. This review primarily focuses on the critical biochemical pathways altered in DR leading to vascular dysfunctions and discusses antioxidants as plausible treatment strategies.

## 1. Introduction

The retina, a transparent tissue of the eye, has an intricate arrangement of neurons and requires highly dedicated circulation to meet its metabolic requirements and functioning of neurotransmission, phototransduction, and complex interaction of metabolites, growth factors, and vasoactive agents. Retinal circulation is a regular geometrically arranged network of vessels with a complex three-dimensional architecture. It mainly is supplied by two vasculatures: the choroid and retinal vessels, where the endothelial cells lining the vessels integrate the normal physiology of the retina [1]. The central retinal artery enters via the optic nerve ensuring blood flow and exchange of gases and nutrients, while the central retinal vein is involved in the removal of waste products that move away from the retina. An important normal physiological function of retinal vasculature is maintenance of the inner blood-retinal barrier (iBRB), which prevents nonspecific permeation of retinal neuropile by macromolecules yet facilitates exchange of respiratory gases, amino acids, salts, sugars, and some peptides [2].

The most sensitive part of the retina is the outer region which constitutes one-third of the retina and is devoid of blood vessels. The absence of blood vessels serves as a special adaptation for visual functioning, but poses a great challenge in the maintenance of continuous energy requirements [1]. The outer blood-retinal barrier that is formed between the tight junctions of retinal pigment cells maintains ionic concentrations in the avascular region of the retina and the interstitial space for neurotransmission. Alternatively, the metabolic need of photoreceptor cells is maintained by choroid vessels. Thus, these efficient blood retinal barriers serve as major anatomical adaptation, with attainment of demanding metabolic requirements of the retina and without compromising its conductive extracellular microenvironment [1, 2]. This intricate retinal vasculature is sensitive to various systemic disorders with diabetes being the most common and perhaps well-studied metabolic insult that has a profound influence on retinal vessels. Retinal vascular dysfunction commences soon after the onset of diabetes and is characterized by impaired microvasculature and transport through the blood retinal barrier which may be an important factor in the initiation and progression of the vascular lesions in diabetic retinopathy [3, 4]. Various studies on diabetes conclude that increased blood flow and impaired autoregulation are key features of diabetic retinopathy [5].

## 2. Diabetic Retinopathy

Diabetic retinopathy is one of the leading causes of visual impairment and morbidity across the globe [6]. Type 1 and type 2 diabetes damages the blood vessels in the retina, which may lead to microvasculature complications; however, the incidence of DR is higher in type 1 patients than in those with type 2 diabetes [7]. Amongst 468 million people estimated with diabetes mellitus worldwide [8], approximately 90 million suffer with some form of diabetic retinopathy, [7].

DR is classified into nonproliferative DR (NPDR) and proliferative DR (PDR) stages on the basis of the presence of visible ophthalmologic changes and manifestation of retinal neovascularization [9]. In the NPDR stage, sex, onset, and duration of type 1 diabetes and HbA1c levels are suggested to be the key pointers implicated in NPDR development [10]. Diabetic maculopathy accompanies the NPDR stage and has been considered as the main reason for the loss of vision. The NPDR stage is primarily consequent to hyperglycemic conditions which weaken the capillary walls resulting in microaneurysms. This is followed by the rupture of vessels which leads to accumulation of fatty deposits and lipid by-products [11]. Ensuing this, an obstruction in the nerve fibre layer is observed that results in white fluffy spots known as cotton wool spots. The NPDR stage ranges from mid, moderate, and severe, where the microaneurysms are followed by venous beading and cotton wool spots along with severe microvascular complications [12].

NPDR is then followed by a proliferative state of the retinal tissue. The PDR stage is a consequence of ischemic conditions that arise due to obstruction in the NPDR stage. The higher metabolic requirement of retinal tissue poses the need for neovascularization which is due to the release of angiogenic signals. Retinal detachment and this neovascularization with the proliferation of the fibrovascular tissue is a characteristic of the PDR stage [13]. These newly formed vessels are leaky, fragile, and misdirected, and with age the shrinkage of the vitreous humour causes them to tear and result in sudden vision loss. If a greater force is created, it may lead to tractional retinal detachment. Despite severe complications in the PDR stage, macular oedema is the main reason behind the loss of visual acuity. Regardless of these varied stages characterising DR, the major progressive change leading to retinopathy in diabetic patients remains speculative in terms of microvascular and biochemical complications leading to oxidative/nitrative stress.

## 3. Microvascular Complication

Microcirculation involves the transfer of nutrients and removal of waste products and regulates the fluctuating hydrostatic pressure of the eye. Normally, fundamental metabolic and myogenic autoregulatory mechanisms guarantee satisfactory advancement of these microcirculatory functions [14]. Microvascular endothelial cells are believed to be targets of hyperglycemic damage since they are not capable of lowering the glucose transport rate when glucose concentrations are high, stimulating intracellular hyperglycemia. This has been thought to be the key event in microvascular endothelial damage, diminished accessibility of nitric oxide, increased permeability, increased leukocyte attachment, and procoagulant action [14].

In one of the recent studies on high sucrose- (HSu-) treated rats, a decrease in the thickness of the inner retinal layers was observed. Nevertheless, neither apoptotic cells nor retinal neural markers were identified in the retinas of HSu-treated animals. Also, no progression was recognized in the permeability of the blood-retinal barrier as well as tight junction proteins. Likewise, these parameters stayed unaltered in the retina regardless of the increase in the number of retinal microglial cells. Thus, a prediabetic rodent demonstrates that the retinal structure is influenced by the diminishing internal layers, without vascular and inflammatory changes [15]. Therefore, inconspicuous auxiliary changes may be seen as an early unsettling influence in the development of DR which could be reversed by preventive strategies at this stage, before it results in irreversible damage to the retina. Progression from diabetes to diabetic retinopathy underlies changes in the haemodynamics or vascular geometry [16]. Haemodynamic factors like perfusion pressure, vascular resistance, blood viscosity, and vascular geometry influence the circulation of blood flow to the retina. Regardless of this fact, the components involved in haemodynamic modifications have not been fully elucidated [14, 16].

In an earlier study, an analysis was performed to assess the relationship between the assessed haemodynamic highlights and the progression of DR. Vessel bifurcations on fundus images and factors like nodal pressure, volumetric blood flow, wall shear stress, blood flow velocity, and Reynolds number were investigated over a period of three years [17]. The analysis revealed critical changes in haemodynamic parameters related with perceptible changes to vessel geometry, especially in the venular network, and these changes were articulated to be three years prior to DR onset [17]. Therefore, these findings altogether suggest a role of the microvasculature and its geometry in the progression of DR, but further studies in this area are yet to be undertaken to fully elucidate the role of altered vascular biology in the progression of DR.

**Figure 1:**
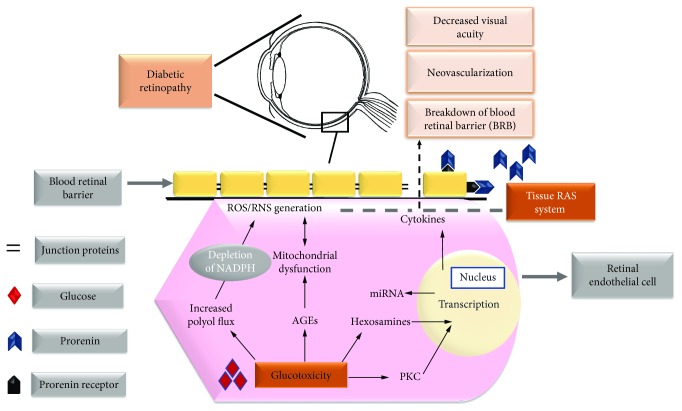
Effect of glucotoxicity on plausible biochemical pathways involved in pathogenesis of DR.

However, vascular modifications are considered to be the major insult for the onset and progression of DR, including altered blood flow, dyslipidemia, basement membrane thickening, loss of pericytes, and platelet aggregation along with neuroglial damage [18]. These changes have been suggested to be the result of alteration/disruption of biochemical mechanisms required for the normal functioning of a cell. A unifying mechanism of hyperglycemia and induced oxidative stress in retinal cells has been regarded as one of the crucial players in causing alteration(s) in various biochemical pathways that have been shown to be interconnected with vascular insult [19, 20]. Furthermore, an increase in the stressful conditions eventually leading to apoptosis of the cells associated with retinal vasculature is the major effect of these pathways. Cardinal biochemical pathways proposed to be involved in the DR includes increased flux of the polyol pathway [21, 22], advanced glycation end product/receptors of advanced glycation end product (AGE/RAGE) pathway [10, 23], hexosamine pathway [24], PKC activation [25], tissue (renin-angiotensin system) RAS [26], and histone modifications which are emerging as key events in the development of DR. The overall effects of these metabolic abnormalities are hypothesized to result in augmentation of (reactive oxygen species) ROS and (reactive nitrative species) RNS production and associated oxidative and nitrosative damage [20, 27] which are key mediators in inducing vascular dysfunctions and related insult to retinal circulation (Figure 1).

## 4. Increased Flux in the Polyol Pathway

Although the polyol pathway is a minor glucose metabolism pathway, it is considered to play a pivotal role in retinopathy [21, 28]. The first and rate-limiting step of this pathway is the conversion of excess glucose to sorbitol, using NADPH as a cofactor, a reaction catalyzed by an enzyme aldose reductase. The sorbitol formed is then converted to fructose by a slow reaction involving sorbitol dehydrogenase [22]. Also, the existence of polymorphism in the aldose reductase gene (C106T) has been shown to be associated with the increased susceptibility to retinopathy in individuals with type 1 (diabetes mellitus) DM [29]. Numerous studies have been carried out to establish the role of increased polyol production in DR. In one of the studies, both rat and human retinal endothelial cells showed increased aldose reductase immunoreactivity. In addition, rat and human retinas exposed to high glucose in organ culture increased the production of sorbitol corroborating excess aldose reductase activity to be one of the mechanisms in the development of DR [30]. Dagher et al. [30] have suggested that the polyol pathway mediates the increase in apoptosis of neurons and attenuation of GFAP- (glial fibrillary acidic protein-) immunostained astrocytes along with the increase in the levels of sorbitol and fructose in the retina. Indeed, this is due to the nonpermeability of sorbitol which results in osmotic damage [21, 31]. Moreover, fructose produced by the polyol pathway gets phosphorylated to fructose-3-phosphate [32], which in turn is broken down to 3-deoxyglucosone; both these molecules are strong glycosylating agents that result in the formation of AGEs. Additionally, the increased flux of the polyol pathway results in depletion of cellular NADPH, affecting the production of reduced glutathione and nitric oxide thereby resulting in antioxidant imbalance [33]. Moreover, it has further been suggested that the polyol pathway is the only mechanism of glucose toxicity responsible for the spectrum of neural and vascular abnormalities [34]. Aldose reductase has been extensively studied as a molecular target for DR, and inhibitors targeting aldose reductase are expected to be beneficial against DR. Inhibitors such as sorbinil and beta-glucogallin (BGG) have been shown to deplete sorbitol accumulation and reduce oxidative stress [33]. Various in silico studies have identified 2-benzoxazolinone derivatives effective against ALR2 (aldose reductase 2) and can reduce AGE and oxidative stress [35]. Additionally, studies on human aldolase reductase have revealed huperzine A, rosmarinic acid, and luteolin 78 to possess aldose reductase inhibition potential [36] and hence could be potential molecules in targeted therapeutics.

## 5. AGE/RAGE Pathway

Elevated glucose and coupled perturbations in the pathways regulating glucose levels result in the formation of advanced glycation end products (AGEs) which are made from nonenzymatic glycoxidation and glycation of various biomolecules and sugar metabolites [37]. AGEs bind to their receptors termed as RAGE (receptor for advanced glycation end product) and trigger the cascade of inflammatory signals [38]. The AGE-modified plasma proteins have been also found to bind to AGE receptors on cells like macrophages, vascular endothelial cells, and vascular smooth cells affecting their functionality [37, 38]. AGEs accumulate in the circulation due to their inefficient renal clearance. Moreover, exogenous AGEs or dietary AGEs have also shown to be the reason for their accumulation in diabetic patients [39] and are considered to be the pivotal participants in inducing the ROS formation by altering the proteins, enzymes, and the genetic material of the mitochondria by glycation [40]. Their accumulation increases the vessel thickening and platelet aggregation leading to ischemic situation, a condition also responsible for the induction of growth factors and neovascularization [41]. Both intracellular and extracellular formations of AGEs in the retina are involved in destructive roles, as the alteration in protein chemistry distorts their structure [39]. Additionally, oxidative stress has also shown to accelerate the formation of the AGEs. Although the human body is self-sufficient in degrading the AGEs by ubiquitination and autophagy, the excess formation or intake results in their accumulation [37]. Additionally, AGEs are also accountable for permanent dysfunction of the mitochondrial enzymes due to glycation of the mitochondrial genetic material leading to “metabolic memory,” a severe condition that is observed in DR [42]. It is an unresponsive state where even glucose control cannot prevent complications of DR. Besides these, the components of the extracellular matrix are subject to modification by AGE precursors.

AGEs have been corroborated to upregulate the expression of RAGE in retinal pericytes via ROS production [43] which is the earliest known alteration in the diabetic retinal vasculature [44]. AGE-RAGE interactions are responsible for NADPH-mediated ROS generation via activation of mitogen-activated protein kinase [38]. These interactions are also responsible for the translocation of NF-*κ*B, decrease in the ratio of Bcl-2/Bax, and increased expression of vascular endothelial growth factor (VEGF), inflammatory cytokines, and adhesion molecules [45], which correlate with the development of DR. During diabetes, AGEs have been shown to accumulate in retinal pericytes, decreasing their survival, breakdown of the blood retinal barrier, and progression towards diabetic retinopathy [9]. Even the major AGE precursor methylglyoxal has been shown to mediate oxidative stress and impair nitric oxide- (NO-) mediated vasorelaxation and upregulate inflammatory markers in an animal model of type 2 diabetes where the rats were fed with methylglyoxal [46]. Also, methylglyoxal in a similar study was shown to reduce retinal pigment epithelial cell viability via ER stress-dependent ROS production, mitochondrial membrane potential loss, and intracellular calcium increase [47]. In a recent study, retinal pigment epithelial cells were treated with chrysin, a naturally occurring flavonoid, to exploit its retinoprotective effects against diabetes-associated visual cycle impairment by targeting the AGE-RAGE pathway. It was demonstrated that chrysin treatment restored the retinoid visual cycle through blocking ER stress via AGE-RAGE activation in glucose-stimulated retinal pigment epithelial cells and diabetic eyes [48], highlighting the importance of the AGE-RAGE pathway in impaired visual cycle associated with diabetic retinopathy.

## 6. Hexosamine Pathway

Another crucial pathway involved in the pathogenesis of DR is the hexosamine pathway which itself is relatively a minor branch of glycolysis where fructose-6-phosphate is converted to glucosamine-6-phosphate, a reaction catalyzed by the first and rate-limiting enzyme, glutamine:fructose-6-phosphate amidotransferase (GFAT) [49]. Chronic hyperglycemia leads to an enhanced influx through the hexosamine pathway which results in perturbation of retinal cells [50, 51]. In this pathway, glucose is metabolized into UDP-N-acetylglucosamine (UDPGlcNAc) [19]. Specific O-GlcNAc transferases (OGT) then modify various cytoplasmic and nuclear proteins by addition of the amino sugar N-acetylglucosamine (O-GlcNAc) from UDP-GlcNAc similar to phosphorylation modification at the ser/thr residues. Pancreatic *β*-cells express large amounts of both O-linked *β*-N-acetylglucosamine transferase (OGT) and O-GlcNAcase (OGA), suggesting the importance of O-GlcNAc in pancreatic *β*-cell functioning and survival under normal glucose conditions [52]. In addition, hyperglycemia-mediated enhanced O-GlcNAc modifications contribute towards increased *β*-cell death [53]. This imbalanced O-GlcNAc modification has been implicated in the etiology of microvascular complications related to DR as it regulates the fate of retinal vascular cells [54, 55]. Moreover, it has been observed that in retinal neuronal cells, this modification alters the neuroprotective effect of the insulin/Akt pathway [56].

Nucleoside diphosphate kinases (NDPK) are the enzymes which provide nucleoside triphosphates to the cells and hence play a pivotal role in mediating fundamental cellular processes [57–59]. NDPKs are histidine protein kinases, which transfer the phosphoryl group from the phosphohistidine active site to the histidine residue of the target protein. These histidine kinases maintain the metabolic status of the cell by regulating the levels of NTP in the cell [57]. The B isoform of this enzyme, NDPK-B, has been shown to phosphorylate the *β* subunit of the G protein, potassium channels (K_Ca_3.1), and calcium channels (TRPV5), thus modulating their functions [60]. Moreover, NDPK-B has been documented in modulating vascular integrity [61]. NDPK deficiency has been shown to mimic vascular regression similar to DR. O-GlcNAcylation of FoxO1, a transcription factor, upregulates Ang 2 (angiopoietin 2) which is an initiator of vascular regression suggesting that the hexosamine pathway is responsible for O-GlcNAcylation of various proteins to be the prime culprit underlying the changes in the molecular signals associated with microvasculature [62]. Corroborating the similar fact, another study has stated the role of NDPK-B deficiency in causing a diabetes-like vascular pathology by upregulating endothelial angiopoietin 2 in the retina of mice [49]. Hyperglycemia increases O-GlcNAcylation of retinal proteins in DR. O-GlcNAcylation of the p65 subunit of NF-*κ*B in streptozotocin-induced DR mice has been shown to be responsible for hyperglycemia-induced activation of NF-*κ*B and retinal ganglion cell death [51], further linking the involvement of imbalanced O-GlcNAc modification in the etiology of the microvascular complications in DR.

## 7. PKC Pathway

Diacylglycerol and PKC are also amongst the key players altered by hyperglycemia in DR [63, 64]. Mainly, three isoforms of PKC are reported in biological systems; viz., the conventional PKC isoforms (PKC-*α*, *β*1, *β*2, and *γ*) are activated by phosphatidylserine, calcium, and DAG or phorbol esters. The novel PKCs (PKC-*δ*, -*θ*, -*η*, and -*ε*) are activated by phosphatidylserine, DAG, or PMA (phorbol 12-myristate 13-acetate), and the atypical PKCs (PKC-*ζ* and -*ι*/*λ*) are not activated by either calcium, DAG, or PMA [65]. In the retina, hyperglycemia persistently elevates diacylglycerol (DAG) and activates downstream protein kinase C, evidently interconnecting PKC with associated microvascular changes [21, 66, 67]. The beta and delta isoforms of PKC are mainly activated, but increases in other isoforms have also been found in the retina [68]. Hyperglycemia activates PKC isoforms indirectly through the AGE-RAGE pathway [69] and polyol pathway [70] by increasing ROS. The DAG-PKC signaling pathway plays roles in vascular cells by regulation of permeability, contractility, extracellular matrix (ECM), cell growth, angiogenesis, cytokine actions, and leukocyte adhesions, processes seen to be altered in diabetes [71, 72]. Numerous studies have demonstrated the contribution of PKC activation in decreasing retinal blood flow. Studies focussed on PKC agonists and antagonists have revealed decreased or increased retinal blood flow, respectively. Introduction of phorbol esters, an agonist of PKC into the retina, declines retinal blood flow while this decrease in blood flow has been shown to be resolved by PKC inhibitors [67].

Amid many targets, a plausible mechanism employed by PKC to cause vasoconstriction and decreased retinal blood flow is the increase in the expression of a potent vasoconstrictor, endothelin A (ET-A) [73]. Its expression has been shown to increase in the retina of diabetic rats while intravitreous injection of the endothelin-A (ET-A) receptor antagonist prevented the decrease in retinal blood flow [73]. This declined retinal blood flow causes hypoxic conditions, which in turn is a robust inducer of VEGF, causing increases in permeability and microaneurysms [74, 75]. *δ* isoform of PKC and p38*α* mitogen-activated protein kinase (MAPK) activation increases the expression of Src homology 2 domain–containing phosphatase-1 (SHP-1), a protein tyrosine phosphatase, which dephosphorylates the PDGF*β* receptor and induces pericyte apoptosis [38, 76]. Previously, it was shown that antiamyotrophic lateral sclerosis (ALS) drug riluzole attenuates pathological changes in oxygen-induced retinopathy, a surrogate model of DR [77]. More recently, a similar study on cultured retinal pericytes as well as in diabetic rats targeting PKC*β* showed that anti-ALS drug riluzole attenuates monocyte chemotactic protein (MCP1), a cytokine elevated in vitreous humour and serum during DR [78] most probably by preventing the abnormal activation of PKC.

## 8. Renin-Angiotensin System

One of the hallmarks and early events of DR is the breakdown of the blood–retinal barrier (BRB), and one of the plausible reasons of this breakdown is renin-angiotensin system- (RAS-) mediated altered vascular permeability [79]. Tissue RAS, a paracrine system, is a property of numerous organs such as eye, brain, vessels, adrenal gland, testis, and kidney, which locally produces angiotensin (Ang) [80]. This system involves prorenin which binds to its receptor, termed as prorenin receptor (P)RR, which has been implicated in the pathogenesis of DR [81] and induces the production of VEGF through ERK1/2, and this signal cascade is known as the receptor-associated prorenin system (RAPS) [82, 83] which is considered to be responsible for the dysfunctional blood retinal barrier. A study on PDR patients has shown the levels of (P)RR to be high in their vitreous fluid samples than in nondiabetic control eyes, strengthening the implication of (P)RR in DR [84, 85]. Also, (P)RR and other RAS system components have also been detected in human PDR fibrovascular tissues, normal ocular tissues, and various human retinal cell lines, including retinal pigment epithelial cells [84, 86], while vitreous prorenin and Ang 2 levels have been reported to increase in PDR eyes [84, 87].

Ang 2, a vasoactive and angiogenic agent, along with VEGF which is a proangiogenic stimulus has been observed to be elevated in the vitreous fluid of PDR patients [88]. Additionally, an increase in VEGF and VEGFR-2 gene expression and an elevation of ocular active renin are indicative of the interaction of tissue RAS and VEGF wherein endothelial cell proliferation is observed as an outcome of the activated tissue RAS system [89], thus correlating the tissue RAS system and VEGF in the progression of DR. In addition, ATP6AP2 or prorenin receptor (P)RR is shown to interact and colocalize with the PDHB subunit of the PDH (pyruvate dehydrogenase) complex. It was observed that PDH activity is downregulated due to ATP6AP2 knockdown, and it leads to suppression of glucose-induced ROS generation in retinal pigment epithelial cells. Thus, ATP6AP2 is considered pathogenic due to its role in RAPS activation and mitochondrial ROS generation [86]. Therefore, blockade of (P)RR and other players of the RAS system might inhibit a series of events vital for vascular abnormalities represented in DR.

## 9. Metabolic Memory and Epigenetic Modifications

Numerous studies have suggested epigenetic modifications to be a significant contributor in DR development [90–92]. The duration of hyperglycemia decides whether improved glycaemic control would be effective in DR [85], implicating that hyperglycemia exposure results in a phenomenon of metabolic memory and could be attributed to epigenetics [91]. Earlier, an anatomic observation of gradual reduction of capillary cells in DR suggested that “retinopathy neither appears promptly after the onset of hyperglycaemia nor arrests promptly on correction of the hyperglycaemia” [90].

The most primitive epigenetic modification is DNA methylation which has a correlation with DR progression as indicated by various studies. DNA methylation is a phenomenon where the methyl group is transferred from S-adenosylmethionine (SAM) to DNA molecules, a reaction catalyzed by DNA methyltransferases. DR patients have shown a significantly higher level of DNA methylation as compared to those without DR [92], indicating that higher DNA methylation is a key component in DR development. Moreover, the study also showed that the levels of DNA methylation remain constant in these DR patients, suggesting that this epigenetic modification occurred only during the early stage of the disease. Another study on DR patients found altered methylated CpG sites, further highlighting the role of epigenetics in DR [93]. Studies using animal models have also strengthened this data where modified methylation patterns have been seen under hyperglycemic conditions [94]. Another study revealed methylation and activation of the matrix metalloproteinase 9 (MMP-9) gene which is known to be associated with DR [95] that plays a role in accelerating the apoptosis of retinal vascular endothelia. In addition, transcription of MMP-9 is regulated by nuclear factor kappa B (NF-*κ*B) whose activation is modulated by the acetylation of its p65 subunit. Histone deacetylase plays an important role in the acetylation-deacetylation of p65. In diabetic mice, histone deacetylase activity was found to be decreased and p65 acetylation was elevated leading to an increase in MMP-9 expression [96].

Another epigenetic alteration, histone modification, has also been a key contributor in DR pathophysiology [97]. The transcription activity of HDAC1/2/8 (histone deacetylase) was elevated in retinal endovascular cells, while the activity of HAT (histone acetyltransferase) and the expression of acetylated histone H3 were both decreased in streptozotocin- (STZ-) induced diabetic models. Moreover, these changes were found to be irreversible after the blood glucose of the rats was restored to normal level, indicating that DR development is to be associated with histone modifications and might be participants in the formation of the “metabolic memory” phenomenon.

Several studies implicated a major role of mitochondrial alteration due to epigenetic modifications as the key process in the induction of metabolic memory in DR. During DR, the mitochondrial homeostasis and dynamics are altered, creating a vicious cycle where the alteration of mitochondrial enzymes induces superoxide formation which in turn alters the organelle physiology. The sensitivity of the mitochondria is exclaimed due to the close proximity of the mitochondrial DNA (mtDNA) to the electron transport chain (ETC) and lack of histones. An increase in 8-OHdG in the diabetic retina confirms the mitochondrial susceptibility [94]. Additionally, the dysfunction of the repair pathways further complicates the mitochondrial damage [98]. The mtDNA replication also plays an important role in mtDNA damage experienced by the retina in diabetes, and these are under the control of superoxide, which is well known to be altered under hyperglycemic conditions. Thus, the regulation of mtDNA replication/repair machinery has the potential to prevent mitochondrial dysfunction and the development of diabetic retinopathy [99].

Histone modifications of the molecules regulating the redox status of the cell has been extensively studied, wherein the mitochondrial superoxide dismutase SOD2 depletion and inhibition of Nrf2 (nuclear factor- (erythroid-derived 2-) like 2), a transcription factor affecting antioxidants, has been observed. During the state of oxidative stress, Nrf2 translocates to the nucleus where it binds to the antioxidant response element (ARE). Keap1, an inhibitor of Nrf2, tethers it in the cytosol and leads to proteasomal degradation through cullin-3-dependent degradation [100]. Mishra et al. [101] have observed that hyperglycemia increased the binding of Sp1, a transcription factor at the Keap1 promoter, and enriched H3K4me1 and activated SetD7 (methyl transferase). This leads to inhibition of Nrf2 binding on antioxidant response element (ARE) leading to oxidative stress in the cell. In earlier studies, deletion in MnSOD (manganese superoxide dismutase) and Sod2 activity *in vivo* has been shown to increase oxidative damage in mitochondria and alters the mitochondrial function [102]. In another study, streptozotocin- (STZ-) induced diabetic rats showed an increase in H4K20me3, acetyl H3K9, and NF-*κ*B p65 at the promoter and enhancer of retinal Sod2. Even the reversal of hyperglycemia failed to prevent increases in H4K20me3, acetyl H3K9, and NF-*κ*B p65 at Sod2. Thus, increased H4K20me3 at Sod2 contributes to its downregulation and is responsible for the development of DR and metabolic memory phenomenon [103].

Dysregulated mitochondrial biogenesis also contributes to the phenomenon of metabolic memory. The nuclear-mitochondrial transcriptional factors and translocation of transcription factor A (TFAM) to the mitochondria are essential for transcription and replication of mitochondria and thus tightly control the biogenesis of the organelle [104]. An earlier study which investigated the effects of diabetes on nuclear-mitochondrial communication in the retina has uncovered that retinal mitochondrial biogenesis is under the control of superoxide radicals and is debilitated in diabetes, perhaps by diminished transport of TFAM to the mitochondria. Hence, regulation of biogenesis by pharmaceutical or molecular means might provide potential means to impede the development/progression of diabetic retinopathy [105]. Additionally, a good glucose control along with lipoic acid supplementation has shown to retard the progression of DR indicative of a major role of mitochondrial function in the progression of disease [105, 106].

## 10. Role of miRNA

MicroRNAs (miRNAs) are a class of 19 to 25 nucleotide bases, noncoding RNAs that regulate gene expression at the posttranscriptional level by annealing to their partially complementary sequences in the target mRNAs resulting in translational repression or degradation of mRNAs, thereby depleting the protein expression [107]. miRNAs have been implicated in the regulation of genes involved in DR development, thus playing a role in epigenetic alterations of DR [108]. A total of 11 miRNAs (miR-182, miR-96, miR-183, miR-211, miR-204, miR-124, miR-135b, miR-592, miR-190b, miR-363, and miR-29c) displayed increased expression in the retinas of DM rats, whereas the expression of 6 miRNAs (miR-10b, miR-10a, miR-219-2-3p, miR-144, miR-338, and miR-199a-3p) was found to be decreased [109]. Moreover, it was observed in cultured human endothelium cells that miR-23b-3p regulates high-glucose-induced cellular metabolic memory through an SIRT1-dependent signaling pathway [110], while in a diabetic rat model, miR-126 was found to play a potential role in the pathogenesis of DR [111, 112]. Additionally, miRNA has also been implicated in regulating retinal neovascularization [112].

## 11. Interplay of Nitrosative Stress/Oxidative Stress and Inflammation

The biochemical mechanisms which are altered in DR probably via hyperglycemic conditions eventually culminate into cellular stress affecting retinal homeostasis. This stressful condition is induced either by the increased flux of these biochemical pathways along with alterations in the proteins involved in maintaining the metabolic energy homeostasis [96]. Also, the mitochondrial complexes are the prime victims due to an increase in reactive oxidative species [96] and nitrative species [113].

### 11.1. Nitrosative Stress

Nitrosative stress is prompted by a response of superoxide with nitric oxide (NO), which creates peroxynitrite and is ensnared in diabetic conditions. Increased nitrative stress could be prompted by protein nitration and damage membrane proteins and fatty acids, prompting changes in cell signaling transduction and upregulation of inflammatory reaction and initiating the apoptotic pathway. Hyperglycemic episodes are connected with increased nitrative stress, which can trigger the advancement in diabetic complications [113].

Mitochondria are additionally equipped for creating both RNS and ROS. NOS exist in three isoforms (endothelial (eNOS), neuronal (nNOS), and inducible (iNOS)) which catalyze the change of L-arginine into citrulline and NO. This response additionally requires flavin adenine dinucleotide, flavin mononucleotide, tetrahydrobiopterin (BH4), heme, and calmodulin. These cofactors are essential (e.g., when BH4 levels are restricted) in light of the fact that NOS may move toward becoming uncoupled and produce superoxide rather than NO. Another plausibility is that, in states of high oxidative stress, NO and superoxide collaborate to produce ONOO−, an exceptionally receptive species equipped for nitrating tyrosine residues, thereby enhancing oxidative damage [113]. The accumulation of RNS shifts the homeostasis of the cell, resulting in nitrosative stress, which is a major phenomenal consequence of the stress conditions in the diabetic retina and is now emerging as a new insight for DR research [113]. NO, a multifunctional molecule that can change proteins by means of nitrosylation, is majorly found in reactive form during DR progression. In addition, nicotinamide adenine dinucleotide phosphate diaphorase (NADPH-d), a specific marker for NO-producing neurons, was revealed to be positive in immunoreactive experiments on diabetic retina suggesting a role of NO in development of DR [114].

Augmentation in nitrosative stress has been revealed as the key player in worsening the pathogenesis of PDR [115]. Additionally, LPO (lipid peroxide), NO, and GSH (glutathione) levels were also shown to be significantly associated with the severity of diabetic retinopathy [116]. An increase of oxidative/nitrative pressure markers, for example, 4-hydroxynonenal and nitrotyrosine, was more elevated in retinal vasculature of diabetic rodents when contrasted with normoglycemic mature and adult rodent retinas [117].

On similar lines, a study showed that the onset of diabetes results in an increase in the nitrated proteins in the retina [114]. Furthermore, nitrotyrosine immunolabelling in the photoreceptor layer, ganglion cell layer, inner nuclear layer, and some Muller cell processes in the retina of diabetic rats points towards the impinging role of nitrosative stress in DR [114]. Conjointly, recent preclinical and clinical studies have indicated a plethora of factors contributing towards the role of NO in the pathogenesis of DR and have pointed towards a further investigation in the plight of therapeutics [118]. Another finding suggests that aminoguanidine treatment hinders capillary cell death and development of diabetic retinopathy [119, 120]. Moreover, it has also been found to decrease the hyperglycemia-induced increase in NO and its sequelae raise a plausibility that (RNS) assumes a major role in the pathogenesis of retinopathy. In any case, it is not conceivable at present to infer that aminoguanidine represses retinopathy exclusively through hindrance of NO generation. Therefore, approaches that specifically repress NO-intervened procedures will be important to absolutely assess the role of NO in the development of DR [121].

Calcium dobesilate (calcium,2,5-dihydroxybenzenesulfonic corrosive (CaD)), which is considered an angioprotective agent, is recommended as an elective treatment for diabetic retinopathy and other vascular diseases, in spite of the debate with respect to its clinical viability. Some clinical preliminaries did not report any helpful impact of CaD treatment, particularly in patients at the later phases of diabetic retinopathy. Notwithstanding, a few different clinical trials demonstrated enhancement in visual acuity after oral treatment. Diabetes increases tyrosine nitration in the retina, fundamentally in the ganglion cell layer, and treatment with CaD attenuates this increase in tyrosine nitration initiated by diabetes [122].

### 11.2. Oxidative Stress

Besides nitrative stress, a shift in the normal physiology due to oxidative stress is also a chief suspect in DR pathophysiology [123]. The balance of oxidants and antioxidants is a mediator of the fundamental cellular process including maintenance of the vascular system [124], which if disrupted can lead to threatening situations depending upon the severity of stress imposed and availability of their clearance system [125]. Unfortunately, imbalance in ROS formation and the scavenging system plays a role in the pathogenesis of diseases including DR as increased oxidative stress has been seen in the retinas of animal models of diabetes [126] as well as in DR patients. In addition, ROS buildup in the eye is considered to be a trigger for degeneration of retinal cells, neural cells, and vascular lesions in the progression of DR. Accumulation of ROS in the eye gradually activates the NF-*κ*B and MAPK cascades that result in inflammation in the retinal tissue. Hence, the interplay of ROS and inflammatory cytokines has been a major area of target and a platform to prevent prognosis of the condition [9]. Along with inflammation, neurodegeneration is another potential target of oxidative stress. Degeneration of retinal ganglion cells (RGC) has been reported to be in close association with oxidative stress and inflammation, wherein the activation of microglial cells results in the neurotoxicity and apoptosis of the RGC. It has been observed that high-glucose free fatty acid cotreatment results in the upregulation of CD11b and ionized calcium-binding adapter 1 (Iba-1), markers of microglial cells, corroborating oxidative species as key players in neurodegeneration, mediated via inflammatory response [127]. Moreover, crocin, a bioactive component of saffron, has been proved to be a good therapeutic agent, due to observed reduction in ROS and NO levels and IL-1*β* and TNF-*α* and additionally with its neuroprotective effect by activating the PI-3/Akt pathway [127]. Another insight into the cause of vascular lesions is neural photoreceptors inducing oxidative stress and inflammatory changes. These photoreceptors are a major source of superoxide radicals (correct this other place also) in the diabetic retina which is due to the contributory effect of both mitochondria and NADPH oxidase. This is further supplemented by the fact that diabetes-induced induction of the proinflammatory molecules iNOS and ICAM-1 was not observed by the removal of photoreceptor cells [128].

Epigenetic changes in the mitochondrial enzymes induced by ROS form a metabolic memory such that even controlled glycemic levels do not respite the symptoms of DR [98]. In retinal endothelial cells, hyperglycemia leads to ROS production, decreasing the levels of a class III histone deacetylase and increasing inflammatory responses from NF-*κ*B [129]. Moreover, this high glucose-mediated ROS production and SIRT1 have been considered important in mediating memory phenomena of retinal endothelial cells [129].

The various species that are involved in oxidative stress includes free radical molecules like superoxide radicals, hydroxyl radicals, nonradicals like hydrogen peroxides and ozone, and reactive lipids like ketosamine and ketoaldehyde groups. These reactive species can be generated by endogenous factors including the electron transport chain of the mitochondria or from the polymorphonuclear cells [130]. Exogenous factors like UV and infrared radiations also contribute to the radical formation. These superoxide species can also lead to formation of peroxynitrate and other reactive nitrogen species, leading to nitrative stress. The eye which allows the light to penetrate every layer makes it susceptible to damage by oxidative or nitrative stress via exogenous factors [131]. Some of the culprit oxidants of DR are mentioned in Table 1. Moreover, oxidative stress also leads to a decline in certain antioxidants thus making it easier for the DR to manifest and progress. These include thioredoxin, superoxide dismutase, NADPH oxidase, Nrf2, vitamin C, and vitamin E and are listed in Table 2.

Although prevention of the progression of retinopathy in diabetic patients lies in the sole strategy of a better diet regimen and maintenance of normal glycemic conditions, new avenues for treatment are increasing with the evolution of better pharmacological targets to treat the severity of retinopathy. Since standard therapies pose a drawback in clinic such as resistance to anti-VEGF intravitreal injection and inflammatory conditions like macular oedema, an hour of need is to find better treatment strategies with lower side effects. Moreover, the balance between prooxidants and antioxidants is disrupted in DR; thus, clinical research involving the use of antioxidant therapies serves as a new direction in relieving the severity of this condition. Some of the antioxidant molecules studied as treatment strategies against DR are mentioned in Table 3. These molecules have shown to be involved in reducing the severity of the disease by exhibiting their effects on the pathways that lead to cellular damage. However, none of the treatments is efficient enough to revert the symptoms completely; further studies are needed to evaluate more potential antioxidants with specific bioactive properties in combating retinal pathophysiology associated with DR.

## 12. Conclusions

**Figure 2:**
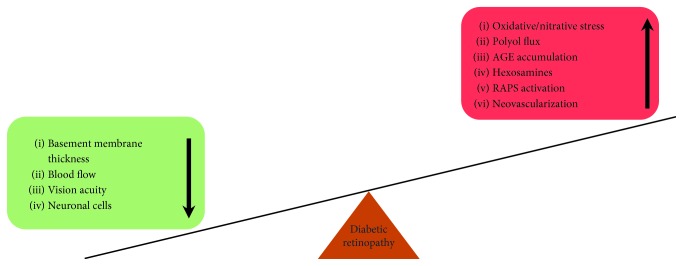
Perturbations in diabetic retinopathy.

Diabetic retinopathy, an acquired blindness, is amongst one of the leading conditions around the globe. The major insult in the progression of DR is the vascular dysfunction in the retinal blood barrier. This is a consequence of the impaired biochemical pathways such as the polyol, AGE/RAGE, hexosamine, PKC, tissue RAS, histone modifications, and altered miRNAs which are considered as major contributors in DR. These perturbations are summarized in Figure 2. The state of metabolic memory has also been implicated along with oxidative stress-induced damage to the mtDNA. This alteration in the mitochondrial complexes is the major inducer of radical species eventually resulting in depletion of antioxidants, leading to inflammation and apoptosis of retinal and endothelial cells during DR. Several treatment strategies aforementioned as the emerging area cardinally target the prooxidant/antioxidant status of the cells to protect from damage in DR. However, more studies are warranted to understand DR development and progression for the benefit of those individuals with retinal damage from diabetes.

## Figures and Tables

**Table 1:** Oxidants elevated in diabetic retinopathy.

Oxidants	Involvement in DR
Superoxide radicals	Accumulation in retinal cell mitochondria leads to mutations in the mtDNA and induces a phenomenon of “metabolic memory” [132, 133].
Hydroxy radicals	Generated by Fenton reaction and responsible for damage to retinal cells membrane and mtDNA as well as reduction of thinning of outer and inner nuclear layers of the retina [133–135].
Peroxyl radical/lipid peroxides	Lipid peroxidation chain reaction damages retinal cell membrane and creates a redox milieu, as evident by an increase in the vitreous of PDR patients [136, 137].
Hydrogen peroxide	Toxic radical species are increased in retinal cells, and treatment with obestatin prevents the H_2_O_2_-induced (retinal ganglion cells) RGC damage [133, 138].
Singlet oxygen	Excitation of oxygen through sunlight and radiation and higher oxygen consumption by retinal cells adds to the impaired redox status of the cell in DR [131].
Peroxynitrite	The reactive nitrogen species shifts the redox status of the cell towards destruction causing apoptosis of retinal endothelial cells [139].

**Table 2:** Antioxidants depleted in diabetic retinopathy.

Antioxidants	Mechanism of action	Effect on DR
Thioredoxin (Trx)	The negative regulator of thioredoxin, thioredoxin-interacting protein (TXNIP), and the dissociation of apoptosis signal regulating kinase-1 (ASK-1) from the oxidised thioredoxin are key players inducing apoptosis during DR [140, 141].	ASK-1 induces apoptosis of Neuro2a cells during DR. TXNIP is upregulated in Muller cells and leads to apoptosis of pericytes [140, 142].
Superoxide dismutase (SOD)	First line of defence against hyperglycemic induced superoxide anion radicals in the mitochondria, and the highest SOD activity is present in the retina to help scavenge the superoxide radicals generated via metabolism [143–145].	SOD downregulation in retinal endothelial cells induces apoptosis [146] and upon overexpression ameliorates and protects the mtDNA from oxidative damage in DR [144].
NADPH oxidase (Nox)	Nox4, an isoform of Nox enzyme, promotes retinal neovascularization through ROS-dependent regulation of the VEGF/VEGFR2 signalling pathway [147] and via various inflammatory signalling pathways [148].	Nox4 isoform gene (*NOX4*) is involved in DR [149] and is the predominant isoform expressed in the human retinal endothelial cells [150].
Vitamin E (*α*-tocopherol)	A nonenzymatic antioxidant which donates hydrogen atom to peroxy radicals and other radicals to maintain the redox status of the cell [151].	Its supplementation reduces oxidative stress in NPDR and PDR patients [152] interlinking an antioxidant role in the prevention of DR.
Vitamin C (ascorbic acid)	An antioxidant or a reducing agent that donates electrons to various radical species [153].	Prevents high-glucose and RAGE-induced apoptosis in pericytes and endothelial cells. Also preserves NO generated by endothelial cells and tightens the leaky endothelial permeability barrier [154].

**Table 3:** Antioxidants as treatment strategies in diabetic retinopathy.

Antioxidant	Effect on DR
*Resveratrol* (RSV)	Reduces ROS levels and cleavage of caspase 3 in BREC (bovine retinal endothelial cells). Additionally, RSV shows antiapoptotic effects *in vitro* and *in vivo* on Muller cells with addition of miR-29b [155, 156].
*Citrus flavones* (hesperidin)	Modulation of mitochondrial function and inhibition of caspase activation via a ROS-dependent p38 and JNK signalling pathway. Also protects retinal pigment cells from hyperglycemic effects [157, 158].
*Citrus flavones* (hesperetin)	Prevents early- or late-stage microvasculopathy by its antiangiogenic properties. Protects Muller cell processes and photoreceptors with an increase in basement membrane thickness in diabetic retina [159, 160].
*Lipoic acid*	Reduces VEGF levels and preserves retinal layer thickness and protects ganglion cells. Also, safeguards injured the retinas of diabetic rats by decreasing oxidative stress, partially via AMPK activation [161, 162].
*Telmisartan*	Increases neurotrophic factors such as BDNF, CNTF, and TH by decreasing caspase-3 activity and increasing GSH levels in the serum and diabetic retina [163].
*Astaxanthin*	Reduces hyperglycemia-induced abnormal proliferation and oxidative stress in retinal pigmented epithelial cells. Also downregulates retinal ganglion cell apoptosis by inhibiting oxidative stress [164, 165].
*Hydrogen sulphide*	Suppresses oxidative stress and exhibits neuroprotective effects on the retina and ablates oxidative stress and inflammation in STZ-induced diabetic rats. However, during PDR stage, increased H_2_S levels are detected in the vitreous cavity and require further studies to understand H_2_S's role in therapeutics [166, 167].
*Tauroursodeoxycholic acid (TUDCA)*	Suppresses inflammatory cytokines and molecules such as NF-kappa B, ICAM-1, and NOS (nitric oxide synthase). Also decreases the levels of VEGF and exerts neuroprotective effects in an experimental retinal detachment model [168, 169].
*Curcumin*	Exhibits hypoglycemic, antioxidant, and anti-inflammatory properties in diabetic rats. Additionally downregulates VEGF and has neuroprotective properties [170, 171].
